# Challenges facing teacher education in Qatar: Q methodology research

**DOI:** 10.1016/j.heliyon.2022.e09845

**Published:** 2022-07-03

**Authors:** Hadeel Alkhateeb, Michael H. Romanowski, Abdellatif Sellami, Abdullah M. Abu-Tineh, Youmen Chaaban

**Affiliations:** a College of Education, Qatar University, Qatar; b Educational Research Center, College of Education, Qatar University, Qatar; c Department of Educational Sciences, College of Education, Qatar University, Qatar

**Keywords:** Teacher education, Teacher challenges, Q-methodology, Qatar

## Abstract

This study aims to identify the challenges facing teacher education in Qatar. Through Q methodology, it examines the ways in which schoolteachers, preservice teachers, teacher education faculty, and Ministry of Education and Higher Education personnel identify what they see as significant challenges faced by teacher education in the country. The overall aim is to provide an overview of teacher education in Qatar and the challenges of improving programs and processes. Results show that the participants' perspectives fall on a continuum of diverse views in which minimal consensus exists. Still, four consensus points were found across the emerged perspectives: schoolteachers’ workload, responsibilities and roles of educational stakeholders, the exasperation towards college-based teacher education, and the impact of culture on teacher education. Based on the results of this study, we argue that these consensus points represent the main challenges facing teacher education in Qatar.

## 1 Introduction

It has been argued that teacher education worldwide is in a crisis (Vanderlinde et al., 2016). Research studies and policy documents report a host of challenges teacher education faces. Some of these challenges relate to the quantity and quality of candidates entering teacher education (De Wever et al., 2016) and the extent to which student teachers attain the required competencies (Valcke et al., 2012). Other challenges arise from the ‘theory-practice gap’ (Korthagen et al., 2001), which refers to ‘the discrepancy beginning teachers encounter between the nature of their teacher preparation program and their experiences as licensed professionals’ (Vanderlinde et al., 2016, p. 9). For some, beginning teachers are poorly prepared (Tait, 2008) and experience anxieties regarding their professional identities (Pillen et al., 2013). Adding to these challenges, policymakers worldwide plea to rethink teacher education to meet the needs of a rapidly changing world (Darling-Hammond, 2017). They constantly broach new requirements, new competency frameworks, new assessment criteria, and new quality indicators that teachers must meet (De Wever et al., 2016).

This study aims to identify the challenges teacher education faces in Qatar through Q methodology. It examines the ways in which schoolteachers, preservice teachers, teacher education faculty, and the Ministry of Education and Higher Education personnel identify significant challenges faced by teacher education in the country. The overall aim is to provide an overview of teacher education in the country and the challenges to improving programs and processes. We first discuss the complex task of preparing teachers, touching upon the literature. Next, we provide a brief outline of the teacher education landscape in Qatar. Then we introduce the Q methodology to investigate the challenges faced by teacher education in the country. Finally, we present and discuss our findings before providing concluding remarks.

## 2 Literature review

### 2.1 The complex task of preparing teachers

Comenius, the seventeenth-century pedagogue, who is considered the father of modern education, stated once that the main challenge of teacher education is ‘to find a method of instruction by which teachers may teach less, but learners may learn more’ (cited in Norlin, 2020, p. 297). Equipping teachers with a flawless method of instruction was and still is a significant challenge in teacher education. However, the challenges facing teachers' preparation today are a different story. Teacher education operates in an increasingly complex context (AASCU, 2016). Scholarly works report and describe a host of challenges teacher education faces in current times (Brown et al., 2015; De Wever et al., 2016; Schleicher, 2018). These challenges may be difficult to address, but they are indeed easy to list.

To begin with, college-based teacher education worldwide has been under severe criticism since its early establishment. Teacher education faculty have long struggled for credibility and legitimacy, both within the university and among public school educators (Zeichner, 2018). This is reflected in Chester's (1991) stern statement that colleges of education ‘are the most-despised institutions in the education universe’ (p. 223). College-based teacher education has even been accused of being a barrier to entry into the teaching profession (Corcoran, 2009). Critics of college-based teacher education often claim that education students are academically weaker than their counterparts in other programs, that education courses are vacuous, that education programs are intellectually superficial, that teacher education faculty are of inferior quality, that colleges of education have meager educational resources, and that educational leadership is insignificant (Zeichner, 2018). Adding to the previous list, college-based teacher education is usually criticized for the ‘theory and practice gap’ (Kinyaduka, 2017; le Velle, 2019; McGarr, O'Grady and Guilfoyle, 2017). That is, teacher education is criticized for not connecting coursework to practice in schools. Darling-Hammond (2009) described this disconnect between theory and practices as ‘the Achilles heel of teacher education’ (p. 8). These criticisms positioned college-based teacher education as ‘a problem’ that needs remedies worldwide (Mayer, 2016). They opened the door, among other factors, for policymakers to interfere.

As college-based teacher education became a policy ‘problem’ that allegedly hindered social and economic development and prosperity, policymakers engaged themselves with identifying the broad parameters that, if controlled and managed correctly, would improve the quality of college-based teacher education (e.g., teacher testing, subject matter requirements, alternate entry pathways, among others) (Cochran-Smith, 2008). Consequently, the global trend of adopting educational standards for preservice teachers and teacher education faculty, accompanied by enforcing notions of accountability and control, has been pushed to ensure quality in many countries (Mayer, 2016). This push for standard-based teacher education often embeds a neoliberal distrust of teacher education faculty and assumes ‘best practice for all.’ Hence, the current policy moment in teacher education has resulted in ‘the reduced professional autonomy of teachers [and teacher education faculty] through prescription, target-setting and evaluation techniques that strip away the subtleties and complexities of the teaching role’ (Storey, 2006, p. 218).

The derogatory depiction and invasion of college-based teacher education contributed to new challenges, especially recruitment. With standard-based teacher education came the demand for fully qualified applicants. Still, unlike other professions, the profession of teaching often endures a shortage of qualified candidates (Wronowski, 2017). This is partially due to the perception of teaching as a low-status profession (Ingersoll and Collins, 2018; Mutluer and Yüksel, 2019; Schleicher, 2018). For instance, in OECD countries, only 26% of teachers agree that their profession is valued in their societies (Ainley and Carstens, 2018).

Increasingly turning into a less glamourized career path, teaching is also fraught with other challenges that drive many teachers to take leave of their profession (Carlo et al., 2013; Ingersoll, 2012). Conspicuous among these challenges is the issue of teaching workloads, which is perceived to impact teacher effectiveness (Salley and Shaw, 2015). Manifested in multiple official tasks, duties, and responsibilities at work, during or after school hours, both inside and outside the classroom, teaching workloads encompass teaching and engaging in bureaucratic paperwork and reporting, curricular and co-curricular activities, and meetings, among other things (Gibson et al. 2015; Stacey, Wilson and McGrath-Champ, 2020). Research highlights concerns about workloads and their impact on the commitment and retention of teachers, a topic that continues to draw policy and decision-making interest (Allen et al., 2020). Indeed, there is evidence suggesting that heavy or overwhelming workloads constitute a crucial factor that adversely affects teachers’ job satisfaction and perturbs their well-being and quality of life (Bridges and Searle, 2011), driving them to feel burnt out (Hester et al., 2020; Bettini et al., 2017). Juggling multiple roles in school has been found to cause teacher attrition (Geiger and Pivovarova, 2018) and reduce organizational commitment, which affects performance at work (Hassan et al., 2018).

In short, it has been argued that the conditions that teachers work in continue to deteriorate (AASCU, 2016). Teachers are asked to do more and meet rising expectations, decrease autonomy, low salaries, tight budgets, and severe teacher shortages in some areas and subjects (AASCU, 2016). This comes with ‘declining enrolment, increasing costs for education majors, difficulties recruiting diverse students and candidates, and shrinking budgets’ (AASCU, 2016, p. 2). All previous challenges are compounded by persistent criticism from policymakers and the media. Additionally, ‘external entities have created policy challenges for teacher preparation programs, including heightened state accountability burdens, unproven regulatory demands, shifts in professional accreditation and burgeoning alternative and emergency certification provisions’ (AASCU, 2016, p. 2). Touching upon literature, the following section examines the place of all these challenges in Qatar's teacher education landscape.

### 2.2 Teacher education landscape in Qatar

Two leading players control and regulate teacher education in Qatar: the Ministry of Education and Higher Education (MEHE) and the College of Education (CED) at Qatar University. The MEHE is the government entity accountable for supporting and regulating education (MEHE, n.d.). It establishes policies and directions for Qatar's education and higher education systems. It works closely with the CED to address the country's K–12 educational needs and Qatar's national priorities. The CED was established in 1973 as the primary provider of teachers to Qatar's government schools (Nasser, 2017). It offers programs in primary education (four concentrations), secondary education (eight concentrations), special education (three concentrations), physical education, and art education (College of Education, 2021). The CED also offers diploma programs in primary education, secondary education, special education, and physical education. Teacher education in the CED reflects a US model in design and structure. In 2016, the CED was accredited by the National Council for Accreditation of Teacher Education (NCATE) and is working to attain accreditation from the Council for the Accreditation of Educator Preparation (CAEP).

Over the past two decades, the landscape of education in Qatar has experienced numerous changes due to educational reforms, and teacher education has been overhauled. Although the MEHE and the CED have hoped for and worked towards attracting and graduating effective teachers, teacher education faces many similar challenges experienced worldwide. Chief among these challenges is the theory and practice gap. Qadhi et al. (2020) reported that teachers graduating from CED struggle to apply theories taught in their teacher preparation programs to classroom practices. In a similar vein, Al-Naimi et al. (2020) maintained that teachers in Qatar's government schools experience ‘a considerable gap between what teachers learned at the College of Education and the reality of the schools’ daily practices' (p. 133). Another challenge faced by college-based teacher education in Qatar is the attraction of male candidates, specifically Qatari nationals (MacLeod & Abou-El-Kheir, 2017; Chaaban and Du (2017). Recruiting male candidates has proven to be challenging for two main reasons. Firstly, males in Qatar have greater access to work in a broader range of higher-status jobs than females (Al-Mohannadi and Capel, 2007). Secondly, the teaching profession in Qatar suffers from being considered low status, and teachers do not receive the desired social status (Al-Khwaiter, 2001; Al-Mohannadi and Capel, 2007; Al-Thani et al., 2021; Lee and Zahir, 2021; MacLeod & Abou-El-Kheir, 2017).

As for teachers' working conditions, it has been argued that teachers at MEHE's schools are operating under increasing pressure. This includes a top-down policy context, a lack of autonomy, heavy teaching workloads, administrative tasks, a lack of administrative support and classroom management challenges (Al-Naimi et al., 2020). Other research studies have demonstrated the difficult working conditions in Qatar's government schools, such as extensive afterschool working hours and work at home, significant government and administrative demands, comprehensive professional development requirements, continual curricula changes demanded by the MEHE, and the forced standardization of lesson plans imposed by the MEHE (Al-Thani et al., 2021; Al-Naimi et al., 2020; Romanowski and Du, 2020; Hendawi, 2020).

These challenges have pushed for the need for more ‘effective’ alternative paths to prepare teachers in Qatar. A particular alternative has been Teach for Qatar (TFQ). In 2014, TFQ is a local non-governmental organization that recruits and prepares graduates and professionals as teaching fellows to teach in Qatari government schools (2019). Two primary narratives explain the emergence of bodies, such as the TFQ. First, there is a cynical narrative that colleges of education have failed and their role in teacher preparation should be reduced (Keller, 2013). Second, it is argued that increasing deregulation and market competition will improve teacher preparation (Zeichner, 2017). Both positions signal a lack of trust in college-based teacher education to graduate quality teachers.

It is important to note that Qatari nationals (333,000) accounted for 10.5 percent of Qatar's total population in mid-2019 (Snoj, 2019). Because of the low number of Qatari nationals, Qatar relies on expatriate teachers in government schools. According to the Planning and Statistics Authority (2019) reports that during the academic year 2017/2018, there were 13,000 primary school teachers (17% Qataris and 83% non-Qataris), 5000 preparatory teachers (13% Qataris and 87% non-Qataris), and 5000 secondary teachers (9% Qataris and 91% non-Qataris). The College of Education graduated 422 female teachers in 2021 (Qatar University, 2021a). Although the number of males and the breakdown of nationality is not available for the 422, one could argue that many of the challenges and issues regarding teacher education and teacher effectiveness are imported. Nevertheless, even if teachers graduated from different universities, the MEHE is still responsible for professional development, and the CED also offers many postgraduate programs that teachers are enrolled. Therefore, all teachers are linked to both the CED and MEHE.

Although, as shown in this section, there is a myriad of research studies that examine the complexity of teaching as a profession in Qatar, there are no studies that we are aware of that conduct a broad analysis of challenges facing teacher education in the country. This study provides that analysis by scrutinizing multiple perspectives of those involved in the preparation of teachers in Qatar. The following section explains how we attempted to do such.

## 3 Methodology

This study uses Q methodology (hereafter referred to as Q) to identify the challenges faced by teacher education in Qatar. Originally, Q was developed based on a simple adaptation of the conventional factor analysis introduced by William Stephenson in 1935 (Watts and Stenner, 2012). Stephenson thought that the conventional factor analytic procedure could be inverted. To clarify, traditional factor analysis studies a selected population of *n* individuals, each measured in *m* tests. Then, ‘the (*m*) (*m* - 1)/2 intercorrelations for these m variables are subjected to… factor analysis” (Stephenson, 1936, p. 344). Instead, Stephenson called for starting with a population of *n* different tests, each scaled by *m* individuals. Next, “the (*m*) (m*-*1)/2 intercorrelations are factorized in the usual way’ (Stephenson, 1936, p. 344). Such an inversion contributed to developing the scientific study of subjectivity (Watts and Stenner, 2012). In concrete terms, the *n* different tests, not research participants, become the study sample in Q research. The variables are no longer tests or hypothesized traits but the research participants themselves (Watts and Stenner, 2012). This allows for exploring ‘correlations between persons or whole aspects of persons’ (Stephenson, 1936, p. 345). Q then is an inverted qualiquantological method (Stenner and Rogers, 2004) that statistically quantifies individuals' subjectivity and offers in-depth qualitative descriptions (Kamal et al., 2014). Q is typically used to reveal the different social perspectives on an issue (Webler et al., 2009). The methodology has proven reliable in several scholarly fields (Watts and Stenner, 2012).

We used Q methods in this study, rather than R methods (e.g., surveys and questionnaires), to achieve two key analytical goals. The first is to explore the perceptions of educational stakeholders regarding the challenges faced in teacher education in Qatar, and the second is to cluster these stakeholders based on their perceptions. For the first analytical goal, while R methods allow research participants the chance to express their views on isolated research statements, which are often constructed by the researcher, Q facilitates the generation of research statements from the participants themselves in various ways (e.g., focus group discussions). Consequently, Q does not enforce a priori meanings on participants; instead, it allows them to express their own subjectivities. In this sense, Q provides a picture of the pre-existing perceptions of research participants (as expressed in opinion articles, for instance) rather than merely scrutinising the level of support for such perceptions (a logic adopted by R methods). For the second analytical task, our goal is to highlight subtle similarities and differences in the stakeholders' perceptions in order to reach a comprehensive social narrative regarding the issue under investigation. In this respect, as Brown (2008) maintained, Q has an established reputation of revealing similarities and differences among individual and group perceptions while capturing areas of friction, consensus, and conflict. In summary, as Coogan and Herrington (2011) remarked, Q is used in this study because ‘no other methods capture the essence of what the participants feel about a topic from collective voices, while at the same time identifying subtle differences between some of these voices’ (p. 25).

Conducting Q research involves four main stages. First, the public argument concerning an issue is sampled to construct what is referred to as a *concourse*. The concourse contains several statements, which are referred to as *Q-items*. Each Q-item expresses a subjective opinion around the issue under investigation. Second, from the concourse, based on careful culling, a representative sample of the Q-items is selected to form the *Q-se*t. Third, participants are chosen strategically and invited to rank the Q-set through a sorting activity, doing a *Q-sort*. Finally, Q-sorts are analyzed using statistical techniques of correlation and inverted factor analysis to reveal underlying patterns among a group of participants. As such, the results are interpreted in the form of different social perspectives (Webler et al., 2009). The application of these stages within this study is detailed below.

### 3.1 Research design

The study begins by developing a representative set of statements that mirror different perspectives on Qatar's teacher education challenges. We utilized an assortment of sources, such as published research studies and newspaper articles (e.g., MacLeod & Abou-El-Kheir, 2017; Al-Mohannadi and Capel, 2007; Al-Khwaiter, 2001; Al-Thani et al., 2021; Lee and Zahir, 2021; Hendawi, 2020; among others). Also, a group of individuals (n = 10) from both the MEHE and the CED (teacher education faculty and preservice teachers) were approached and invited to two focus-group sessions. These individuals were asked to provide feedback concerning the challenges facing teacher education in Qatar. Specifically, they were asked to respond to the following questions:

1. What are some of the main challenges facing teacher education in Qatar?
2. What are the problems that teachers face in Qatar?
3. How do you think the main challenges facing teacher education in Qatar can be overcome?
4. What should be done to overcome the problems teachers face in Qatar's teaching profession?

Responses to the previous questions were recorded in an Excel file. This collection gradually grew to nearly 3,500 words. This body of responses constituted a range of subjective opinions for this study: the concourse. Next, we needed to reduce the concourse to a Q sample that is ‘small enough for practical purposes and sufficiently diverse to approximate the diversity of the concourse’ (Brown et al., 2018, p. 3). Our initial impulse was to structure the concourse around three main categories*: CED and teacher education, MEHE and teacher education, and culture and teacher education*. This resulted in 41 statements, which were culled with great attention to discard repetitious and marginal statements (Lo Bianco, 2015). This resulted in 29 Q-items. These items were then reformulated in Arabic, as it is the native language of the potential participants. Next, each Q-item was specified by a number to identify the statement for numbered data recording. Next, we piloted the culled items with two people: a senior employee from the MEHE and an academic faculty from the CED. This resulted in some modifications. Then the Q-set was completed. Table 1 illustrates the Q-items corresponding to the three main categories mentioned earlier, while Appendix 1 lists the Q-items.

**Table 1:** **Q-items categories**.

Category	Q-items
CED and Teacher education	Q-items: 12, 13, 14, 15, 16, 17, 18, 19, 20, 21, 23 and 24
MEHE and Teacher education	Q-items: 1, 2, 3, 5, 6, 7, 8, 9, 10, 11 and 22
Culture and Teacher education	Q-items: 4, 25, 26, 27, 28 and 29

### 3.2 P-set

Watts and Stenner (2012) argued that ‘perhaps the most important single message about participant recruitment in Q methodology is that opportunity sampling is rarely the best strategy’ (p. 70). This is mainly because, as mentioned earlier, Q represents an inversion of R methodological techniques. In Q research, participants serve as variables, while the Q-set constitutes the study sample. This means that ‘strategic, rather than opportunity, sampling of participants is preferable’ (Watts and Stenner, 2012, p. 88). Also, a comparatively small number of participants, relative to the R method, is desirable in Q research. As Watts and Stenner (2012) noted, ‘Q does not need a large number of participants, and it is not interested in headcounts. It just needs enough participants to establish the existence of its factors’ (p. 88). This is reflected in several educational studies that have adopted Q as a research methodology. For example, Mesci and Cobern (2020) used Q to examine six middle school science teachers' understanding of the nature of science, while Ramlo (2015) used Q methods to determine the perceptions of 22 students of a flipped classroom experience. Similarly, Dariel et al. (2012) used the methodology to explore the underlying factors influencing the learning of 38 e-learners, and Alkhateeb and Alshaboul (2021) examined 16 homeroom teachers' understanding of the importance of a student's mother tongue in international English-medium primary schools. In short, as Brown (1980) maintains, in Q research, ‘all that is required are [sic] enough subjects to establish the existence of a factor for purposes of comparing one factor to another’ (p. 355). Participants should also have a defined viewpoint to express and, even more importantly, their perspectives should matter in relation to the issue under investigation (Watts and Stenner, 2012).

Based on the previous criteria, 69 participants were recruited to undertake Q-sorting activities. These participants came from the following groups: MEHE personnel, teacher education faculty, schoolteachers, and preservice teachers. The participants received (1) a clarification about the study, (2) a consent form, and (3) a blank sorting grid. They were asked to sort 29 Q-items on a scale of +4 to −4 in response to the question (i.e., the condition of instruction, in Q terminology): *Rank each statement based on the level of agreement or disagreement.* The grid was created as a 9-point forced quasi-normal distribution, as this kind of distribution facilitates the revealing of perspectives (Brown, 2008). The farthest right column represented ‘most agree’ (+4), while the farthest left column represented ‘most disagree’ (−4), and the middle column was considered ‘neutral’ (0). Upon completing the sorting activity, the participants were asked for an explanation for their selections regarding each item labeled +4 or −4. These explanations were transcribed and later analyzed. Table 2 shows the P-set of this study.

**Table 2:** P-set.

Category	Number
MEHE personnel	10 participants
Teacher education faculty	15 participants
Schoolteachers	11 participants
Preservice Teachers	33 participants
	**N = 69**

### 3.3 Statistical analysis

PQ-Method software was used to statistically analyze the data gathered from the 69 participants. We first performed a centroid factor analysis, a factor extraction procedure that looks for repeated patterns by performing a by-person factor analysis. This was followed by conducting a varimax rotation to account for the maximal amount of opinion variance (Watts and Stenner, 2012). Based on statistical criteria (e.g., the eigenvalues) and the researchers' intuitions (Watts and Stenner, 2012), three factors (F1, F2, and F3) were extracted. Each of these factors represents a social perspective shared by a group of participants and all combined account for 35% of the opinion variance. For Kline (1994), any solution explaining 35–40% of the study variance is sound. Next, Brown's equation (1980) was used to calculate each Q-sort's significance at the p < 0.01 level: 2.58 × (1 ÷ √no. of items in the Q-set). Factor loadings of at least ±0.48 were significant at the p < 0.01 level in this study.

**Figure 1:**
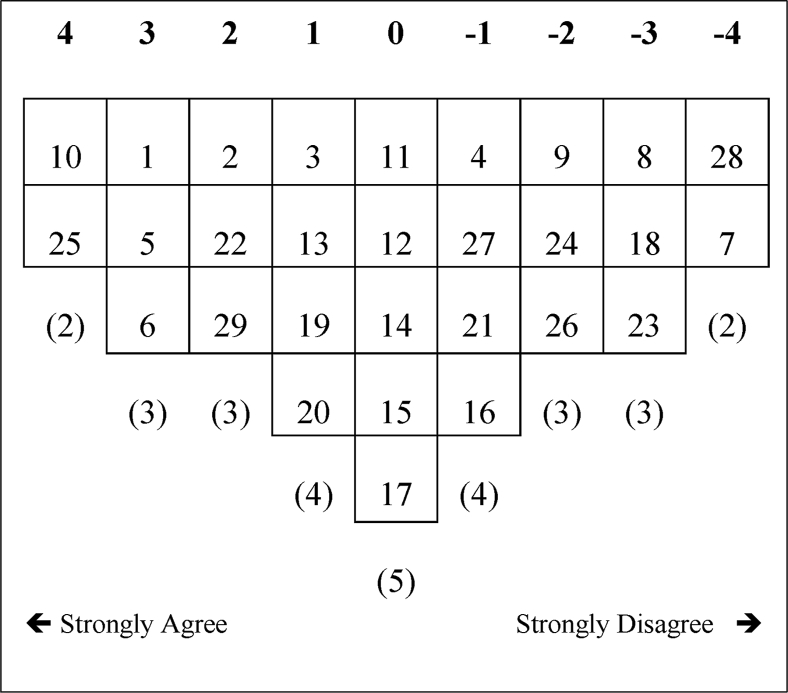
Factor array for F1.

**Figure 2:**
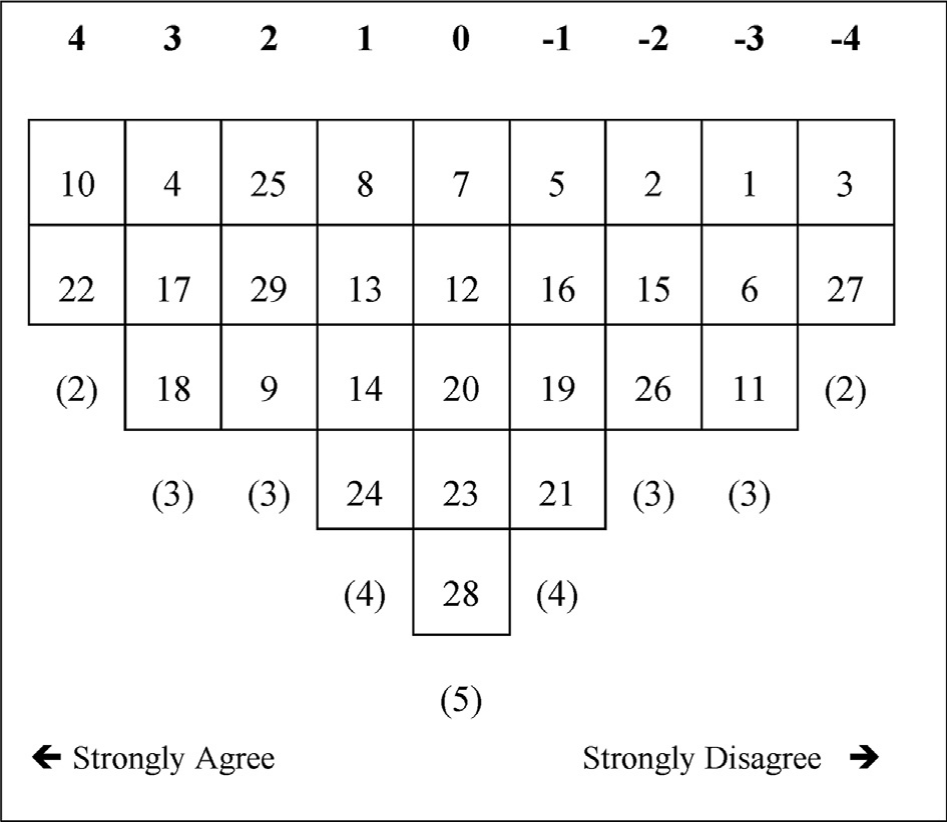
Factor array for F2.

**Figure 3:**
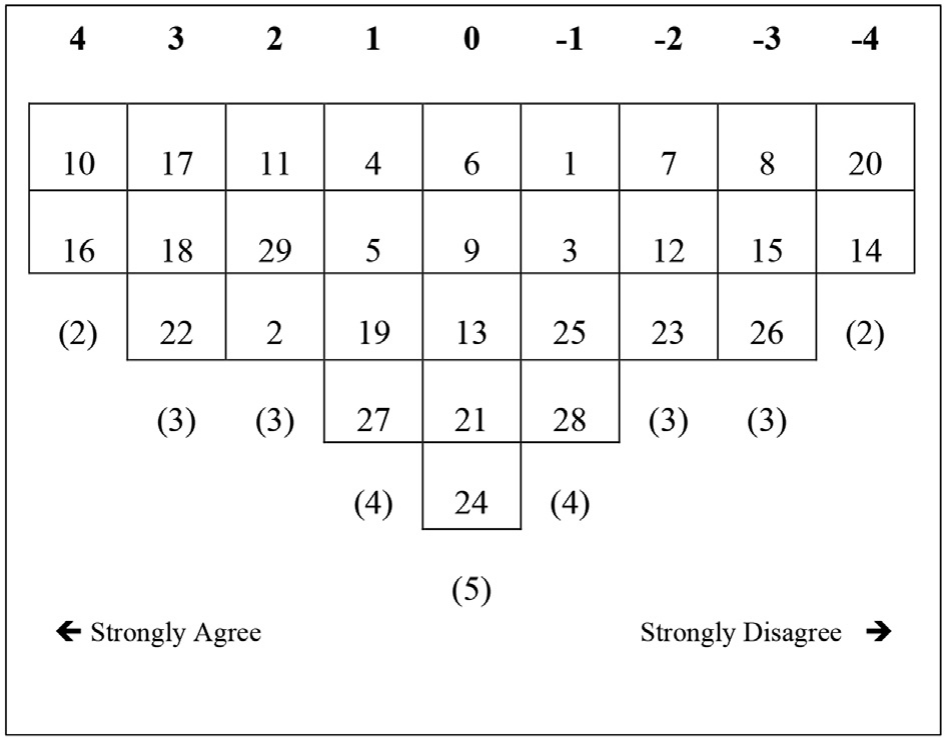
Factor array for F3.

A total of 53 of the 69 Q-sorts loaded significantly on one of the three emerging factors, while 16 were null cases. All Q-sorts loaded on the same factor emerged to a signal ideal Q-sort. Such a shared understanding of the issues under investigation is referred to as a *factor array*. Figures 1, 2, and 3 show the factor arrays for the emerging factors in this study.

## 4 Results

As previously mentioned, three factors (F1, F2, and F3) were extracted; each represents a social perspective on the challenges facing teacher education in Qatar shared by a group of participants. Table (3) quantitively summarizes all the emerging factors, including the opinion variance and significant loadings, while Q-sort values for items are presented in Appendix 1 (Table 3).

**Table 3:** Emerging factors.

Factor	F1	F2	F3	Null	
Number of loadings	22	17	14	16	N = 69
% Explained variance	15	11	9		

We qualitatively present the three emerging factors (F1, F2, and F3) in the coming sections. Each factor was given a label representing its general sentience (Stenner and Rogers, 2004). These labels are as follows:

**F1:** We blame the carpenter, the MEHE.
**F2:** We blame the carpenter's tools; the CED.
**F3:** We blame the carpenter and the tools; the MEHE and CED.

Through our qualitative interpretation, we employ Q-items and participant comments during the sorting activities. The figures in brackets represent Q-item ranking. To illustrate, for F1 (Q-item 10: +4) implies that Q-item 10 was ranked in the most agreeable position by the merged average of all participants loaded on this factor, whereas (Q-item 7: -4) suggests that Q-item 7 was placed in the most disagreeable position.

### 4.1 Factor 1: we blame the carpenter; the MEHE

Twenty-two participants (comprising two MEHE personnel, four schoolteachers, four teacher education faculty, and twelve preservice teachers) loaded on this factor. Participants here believe that the MEHE's ways of governing schools hamper teachers' willingness to join the profession (Q-item 1: +3). For them, in a manner similar to carpenters who depend on raw materials (wood/timber) to construct, install, and repair structures, the MEHE needs to hire and professionally develop CED graduates, relying on their knowledge and competencies. However, the situation is rather complicated. The relationship between the MEHE and CED is not up to expectations and requires improvement and collaboration (Q-item 22: +2).

Participants loaded on this factor blamed the ministry's ineffective leadership (Q-item 5: +3). They strongly believe that schoolteachers' large amount of work hampers the teaching profession (Q-items 10: +4). Hence, the working conditions in government schools do not attract CED graduates (Q-item 7: -4). Moreover, the sudden forced change from teaching in one school to another that schoolteachers face hinders schoolteachers' willingness to stay in the profession (Q-item 6: +3). Participants here believe that teaching methods in government schools are forced on schoolteachers (Q-item 2: +2) and that the relationship between principals and schoolteachers is not at its best all the time (Q-item 9: -2). To make matters worse, for participants loaded on this factor, the MEHE denies schoolteachers many privileges and benefits (Q-item 8: -3). Perhaps this, among other factors, led to a negative view of teaching as a career in Qatar, as participants here strongly believe (Q-item 25: +4) and prevented society from creating and building a culture of innovation in the teaching profession (Q-item 28: - 4). After all, for participants here, the Qatari conservative culture drives certain groups, mainly women, to become schoolteachers regardless of their genuine interests (Q-item 29: +2). Without a genuine interest in teaching as a profession, the transition from high school to the CED is a difficult adjustment for participants loaded on this factor (Q-item 23: -3). With all these complications, the CED cannot perform well as a teacher education provider (Q-item 26: -2). In short, participants loaded on this factor seem to relate the challenge of graduating and recruiting effective teachers in Qatar with the MEHE's presumed poor management and unsuccessful policies.

### 4.2 Factor 2: we blame the Carpenter's tools; the CED

Seventeen participants (six of MEHE's personnel, four schoolteachers, two teacher education faculty, and five preservice teachers) loaded on this factor. On the one hand, these participants believed that teacher education in Qatar is inadequate. The basis of their belief is that the CED is not performing well as the main national provider of teacher education (Q-item 26: -2). Participants here believe that the CED's management impedes the aim of graduating effective teachers in various ways (Q-item 18: +3). Chief among them is recruiting teacher education faculty of inferior quality (Q-item 17: +3). Consequently, preservice teachers do not receive effective learning experiences or valuable field training (Q-item 15: -2). Besides, for participants loaded on this factor, the CED's enrollment criteria should be revised (Q-item 13: +1), as education is an easy degree at Qatar University (Q-item 24: +1). For all of this, participants here strongly believe that collaboration between the CED and MEHE is needed now more than ever (Q-item 22: +4).

On the other hand, participants loaded on this factor vigorously defended the MEHE's policies and management. They were vehement in dismissing any criticism targeted at MEHE's educational governing, which, according to some people, could decrease individuals' willingness to join the teaching profession in the country (Q-item 1: -3). Additionally, participants here strongly refuted the common perception that the MEHE has an adequate supply of outsourced, non-Qatari teachers who receive lower salaries than citizens. As a result, they believe there is no genuine interest or need to prepare national schoolteachers (Q-item 27: -4). For participants loaded on this factor, the MEHE spares no effort to attract, prepare, and professionally develop national teachers. This includes ensuring good working conditions for schoolteachers. Hence, participants here believe that there is a good relationship between principals and schoolteachers in schools (Q-item 9: +2).

Additionally, the teaching methods in government schools are neither traditional with little room for innovation (Q-item 3: -4) nor forced on schoolteachers (Q-item 2: -2). Similarly, participants here refused to consider the unclear teacher transfer policies as negatively influencing teachers’ willingness to join and stay in the profession (Q-item 6: -3). They also did not see the frequent changes in curricula in government schools as a problem in preparing teacher graduates (Q-item 11: -3).

Still, why the Qatari government invests in programs to attract nationals to become teachers, but few continue in the profession is strongly present in the minds of participants loaded on this factor (Q-item 4: +3). For them, a likely answer could be the amount of work that teachers must do in government schools (Q-item 10: +4). Conversely, participants here believed that role of teachers has changed from being a sage on the stage to becoming a facilitator of learning. This change necessitated more effort and commitment. Another possible answer could be the negative view of teaching as a career in Qatar (Q-item 25: +2) and the conservative Qatari culture that drives certain groups (mainly women from conservative families) to become schoolteachers, regardless of their genuine interest (Q-item 29: +2). To conclude, participants, loaded on this factor, scapegoat mainly the CED and partially the local culture, with the challenge of attracting, preparing, and retaining effective teachers in Qatar.

### 4.3 Factor 3: we blame the carpenter and the tools; the MEHE and the CED

Fourteen participants (one MEHE personnel, two schoolteachers, five teacher education faculty, and six preservice teachers) loaded on this factor. For these participants, both the MEHE and the CED need to assume responsibility and act accordingly to face the country's challenges in teacher education. To begin with, participants here strongly believe that the CED's curriculum is outdated, which is a challenge to graduating distinguished teachers (Q-item 16: +4). The participants assume this is coupled with a lack of high-quality teacher education faculty (Q-item 17: +3) and ineffective management (Q-item 18: +3). Hence, for participants loading on this factor, the CED is struggling to prepare preservice teachers with the best educational practices (Q-item 20: -4), which involves providing them with valuable field training experience (Q-item 15: -3) and a life-long ability to develop as experienced teachers after graduation (Q-item 14: -4). As such, participants here believe that the CED, as the primary national teacher education provider, is not performing well (Q-item 26: -3).

With similar emphasis, participants loaded on this factor believe that the working conditions in the schools intimidate individuals wishing to join the teaching profession (Q-item 7: -2). The main issue is that the immense workload of teachers in government schools hampers the teaching profession (Q-item 10: +4). Concurrently, the MEHE denies the deserved privileges and benefits for schoolteachers (Q-item 8: -3) and forces teaching methods on them (Q-item 2: +2), with frequent disturbing changes in curricula (Q-item 11: +2). From the participants' standpoint, the adverse outcomes of such situations include the loss of dedicated schoolteachers, the burnout of distinguished teachers, which reduces their effectiveness, and the decreasing interest of some individuals in entering the teaching profession. In this regard, participants loaded on this factor believe that the relationship between the CED and the MEHE needs improvement and better collaboration (Q-item 22: +3). In short, participants here believe in the shared responsibility and accountability between the MEHE and the CED for shared prosperity in the country's teacher education.

## 5 Discussion

This study aimed to identify the challenges faced by teacher education in Qatar. Utilizing Q as a research methodology, we examined the ways through which schoolteachers, preservice teachers, teacher education faculty and MEHE personnel identify the perceived significant challenges found in Qatar's teacher education field. Overall, the results show that the participants' perspectives fall on a continuum of diverse views in which minimal consensus exists. Still, four consensus points were found across the emerged perspectives. In the next section, we discuss these points, which we argue, based on the data of this study, are the main challenges facing teacher education in Qatar.

### 5.1 Challenge one: schoolteachers’ workload

Of all the emerging factors, participants unanimously agreed on Q-item 10, which was concerned with the adverse effects of teachers' excessive workload on the teaching profession in Qatar. Perhaps of even more interest was the consensus reached by participants (+4, +4, +4) across all emerging factors. Some participants elaborated on the unnecessary and unproductive tasks that take up too much of the teachers’ time during the Q-sort activities. Participants mentioned issues such as the high number of classes that schoolteachers teach daily, which leads to time-consuming planning and excessive marking and reporting. Additionally, schoolteachers are often involved in administrative tasks and the implementation of frequent changes in curricula and policies. Participants, mainly schoolteachers, reported a sense of being on an educational pendulum, swinging back and forth all the time. When the pendulum swings one way, schoolteachers work hard to invest in materials and resources that align and support this, but when some of the imposed policies or initiatives do not work, the pendulum swings in a different direction. Schoolteachers feel burnt out by the amount of work these changes take. They face this pressure while also undergoing a continual reduction of their annual vacations and a lack of proper incentives, negatively affecting their well-being and quality of life. In the worst-case scenario, this may lead to schoolteachers leaving the profession. Perhaps more critical is that participants—mainly schoolteachers—reported feeling compelled to complete the required tasks and guilty if they did not deliver them, leading to an extra emotional burden.

There is evidence that MEHE is aware of the substantial workload placed on schoolteachers. Local media reported that the MEHE distributed a circular to government school principals requesting that the administrative tasks assigned to teachers be reduced (Al-Sharq, 2021). The circular recommended that principals decrease schoolteachers' workload to allow them to focus on their primary job of teaching students. Schools conducted meetings, resulting in recommendations to accommodate the MEHE circular. However, as the data of this study shows, practices still vary on the ground. Such a challenge could be mitigated if clear expectations of schoolteachers’ roles were defined and emotional support, including advice, counseling, and coaching, was provided.

### 5.2 Challenge two: responsibilities and roles of educational stakeholders

Across all the emerging factors, participants unanimously agreed on Q-item 22, which articulated the need to enhance the coordination between the MEHE and the CED, the two main pillars of Qatar's teacher education. Q-item 22 received strong positive scores in the three emerging perspectives (+2, +3, +4). Research suggests that coordination between educational stakeholders tends to be limited or infrequent (European Commission, 2013). Schoolteachers are likely to have little contact with teacher education faculty, who may also rarely interact with each other or with teacher education recruiters. This lack of coordination within teacher education can go even deeper: teacher education faculty themselves may adhere to different professional values, which may even oppose teacher education recruiters' standards (European Commission, 2013).

In some countries (for example, the Netherlands), associations of teacher educators promote coordination, communication, and dialogue between the different stakeholders. Such associations play a critical role in addressing issues of common interest, launching professional dialogue, and informing the development of policy and legislation. Where professional associations are absent, teacher education is fragmented over various institutional contexts. In such circumstances, teacher education may face considerable challenges in ensuring consistency and quality in its content and delivery. Hence, as the data of this study shows, those involved in teacher education may become disappointed and frustrated, sensing the existing problems.

It has been argued that ‘the key [educational] stakeholders … need to be involved in decisions about the teacher education. It is important that they achieve consensus on a shared vision—with a common understanding of what is meant by quality in educating teachers—and the actions needed to support teacher educators’ (European Commission, 2013, p. 11). To develop such a collective understanding, it can be useful for all educational stakeholders to meet and discuss aspects of their profession beyond institutional borders. This can occur in formal structures, such as professional associations, and informal contexts, such as networks and communities (European Commission, 2013). Concretely, cultivating quality in teacher education is easier to attain when there is collaboration between all the key actors. This necessitates an active and continuous professional dialogue between all educational stakeholders, leading to shared understandings and expectations about the aspired teacher education.

### 5.3 Challenge three: the exasperation toward college-based teacher education

An area of ‘consensus against’ across all the emerging perspectives was Q-item 26, which stated that the CED was performing well as the main national provider of teacher education. All participants gave negative scores to this statement (−2, -2, -3). Yet, it is worth noting that the reason behind the negative scoring differed among each of the emerging perspectives. For participants loaded on factor F1, the CED performance was seen as poor due to ineffective MEHE policies, leadership, and lack of communication (see Appendix 1 for F1's participants' loadings on Q-items 7, 10, 6, and 2). However, the case in F2 was different. Participants loaded on F2 demonstrated a deep mistrust of college-based teacher education represented by the CED. For participants loaded on F3, it could be argued that the result came from a combination of both beliefs: a disappointment with MEHE leadership and dissatisfaction with CED outcomes.

Kennedy (1998) argued that criticism and skepticism of the effects of teacher education are not novel. Since its beginnings, college-based teacher education has always been questioned regarding its value and effectiveness. Zeichner (2018) suggested that the media often played a role in constructing and promoting a ‘narrative of failure’ (p. 106) about college-based teacher education. Such a narrative often inflates the public perception of these organisations ‘beyond what is warranted by the available evidence’ (p. 106). It seems that ‘everyone has an opinion about what effective teacher education looks like, often anecdotally informed or politically motivated’ (Mayer, 2016, p. 33).

Grossman (2008) admitted that ‘as researchers and practitioners in the field of teacher education, we seem ill-prepared to respond to critics who question the value of professional education for teachers with evidence of our effectiveness’ (p. 13). Hence, the challenge faced by college-based teacher education is to provide evidence of its effectiveness. One way to do this is by directing research to questions about the value of teacher education. There are some examples of such research from different parts of the world (e.g., Louden et al., 2010; Brouwer and Korthagen, 2005; Hobson et al., 2009; among others). Such research has responded to teacher education critics through large-scale empirical studies employing mixed methods, including ethnography. This kind of research is still absent in the Qatari context. However, as Wiseman (2012) reminds us, allegations that ‘teacher education is failing us’ will continue. Hence, ‘teacher educators must be prepared to participate in the debates in an informed and reasoned manner’ and ‘it will be up to us [teacher education faculty] to contribute scholarly solutions to the policy questions and issues’ (p. 90).

### 5.4 Challenge four: the impact of culture on teacher education

Across all the emerging perspectives, participants gave positive scores to Q-item 29 (+2, +2, +2), which accuses Qatar's conservative culture of tracking particular groups (i.e., females from conservative families) to become teachers without paying any regard to their real interests. Past research shows that women's participation in segregated female schools and workplaces ‘fit [s] into the previously existing social arrangements’ and does not ‘pose a challenge to the dominant conservative ideology regarding the definition of the boundaries of woman's world’ (Hatem, 1985, p. 103). The recent scholarship supports the claim that a conservative ideology in the region often can place boundaries on women's choices of career paths (Jayachandran, 2021; Miller et al., 2020).

Despite the relatively recent changes sweeping across Arabian Gulf societies, attributed primarily to the forces of globalization and modernization, social practices and cultural values remain dominant factors influencing women's occupational choices in these patriarchal societies and limit the sectors in which they desire to work (Ensour et al., 2017). Concerns about Qatari women working in a mixed-gender environment are not uncommon. Women's participation in the workforce exhibits a visible concentration in a limited number of professions: education, health care, and clerical jobs (Stasz et al., 2007).

Interestingly, official statistics reported in Qatar University's Book of Trends (2021b) indicates a significant gender gap in male-female Qatari student enrollment in the CED. For example, for the current academic year (2020–2021), the undergraduate student cohort consists of 2,016 Qataris (72 males and 1,944 females). A mere 35 Qataris make up the master's degree cohort (2 males and 33 females). The question then becomes, should the gender imbalance in Qatar's teaching profession be a priority for both the MEHE and the CED, requiring further research and ultimately a solution? McGrath et al. (2020) provide insights into why the answer to the question should be ‘Yes, indeed!’ McGrath et al. (2020) offered four reasons why male teachers are needed in the teaching profession: At the child level, the ‘limited observation of male teachers may result in children's erroneous generalization of all teacher characteristics as female-specific traits, perpetuating the view that women are better suited to the teaching profession’ (p. 6). McGrath et al. claimed that male students benefit from relationships with male teachers at the classroom level. At the organizational level, the authors emphasized the benefits of a representative workforce on interactional experiences and policy development. Finally, greater male teacher representation may challenge fixed social constructions of masculinities, femininities, and stereotypical gender roles at the societal level.

## 6 Conclusion

Through Q methodology, we examined how educational stakeholders identify the main challenges faced by teacher education in Qatar. The aim was to provide an overview of teacher education in the country and the challenges of improving programs and processes. Although results show that the participants' perspectives fall on a continuum of diverse views, four consensus points were found across the emerged perspectives. These are, as mentioned earlier, schoolteachers' workload, roles of educational stakeholders, the exasperation towards college-based teacher education, and the impact of culture on teacher education. Some of these challenges are similar to those in other parts of the world. That is, relevant global research highlighted challenges such as schoolteachers' workload (Kokkinos, 2007; Tsang, 2019), the crucial relation between educational stakeholders (European Commission, 2013), and the exasperation towards college-based teacher education (Grossman, 2008; Zeichner, 2018). Still, some of these challenges make the Qatari situation peculiar. Mainly the impact of the country's conservative ideology on teacher education. In this regard, we argue that more empirical research is needed to describe and explain the implications of prevailing societal ideologies on recruiting and preparing teachers in Qatar and elsewhere.

We conclude this study by pointing out the most pertinent goal of teacher education-producing well-prepared schoolteachers. However, achieving this goal mainly depends on understanding how different stakeholder groups view and interact with one another within the complex system of teacher education. It has also been said that how teachers should be prepared and recruited is relentlessly contested by those involved in delivering teacher education and by external critics who consider teacher education to be a straightforward process that leads directly to desired educational outcomes (Hess and McShane, 2014; Kumashiro, 2015). In closing, we emphasize the complexity of teacher education systems. A common approach to teacher education research has been to simplify teacher education's challenges by focusing on a few critical parts of the system without necessarily addressing how the parts are related to and influenced by other parts and factors (Ludlow et al., 2017). Instead, as Opfer and Pedder (2011) proposed, researchers should resist simplification and reduction and should conceptualize teacher education as a complex overlapping system, thus developing explanatory theories that incorporate the multiple perspectives, nested contexts, processes, and nonlinear relationships of the system's elements (Ludlow et al., 2017). We anticipate that this research—and similar scholarly endeavors—will pave the way for empirical investigations that draw on network and systems theory strategies to reveal the group-level perceptions, the within-group heterogeneity, and homogeneity, and the shifting between-group perceptions of all those involved in teacher education regarding their roles and the challenges faced. Ultimately, the findings of such investigations will enhance systems of teacher education, which in turn will feed back into the development of the next generation of prepared teachers.

## Declarations

### Author contribution statement

Hadeel Alkhateeb: Conceived and designed the experiments; Performed the experiments; Analyzed and interpreted the data; Wrote the paper.

Michael H. Romanowski; Youmen Chaaban: Conceived and designed the experiments; Analyzed and interpreted the data; Wrote the paper.

Abdellatif Sellami: Analyzed and interpreted the data; Wrote the paper.

Abdullah M. Abu-Tineh: Conceived and designed the experiments; Performed the experiments.

### Funding statement

This work was supported by Qatar National Research Fund [National Priorities Research Program (NPRP13S-0209-200319)].

### Data availability statement

The authors do not have permission to share data.

### Declaration of interests statement

The authors declare no conflict of interest.

### Additional information

No additional information is available for this paper.

## Figures and Tables

**Table:** 

Q-item	F1	F2	F3
1. The MEHE's traditional ways of governing schools, hampers teachers' willingness to join the profession.	3	-3	-1
2. Teaching methods in government schools are forced on teachers.	2	-2	2
3. Teaching methods in government schools are traditional, with little room for innovation.	1	-4	-1
4. The government invests in programs to attract Qataris to become teachers, but few continue in the profession.	-1	3	1
5. The MEHE's leadership is a challenge for teacher education.	3	-1	1
6. The sudden forced change from teaching in one school to another that schoolteachers face hinders their willingness to stay in the profession.	3	-3	0
7. The working conditions in the government schools attract CED's graduates.	-4	0	-2
8. The MEHE provides many privileges and benefits for teachers.	-3	1	-3
9. In a general sense, there is a good relationship between principals and teachers in government schools.	-2	2	0
10. The large amount of work that teachers have to do in government schools hampers the teaching profession.	4	4	4
11. The frequent changes of curricula in government schools make it difficult for teacher education to prepare teachers.	0	-3	2
12. The low quality of students entering the teaching profession in the CED is a challenge to the teaching profession in Qatar.	0	0	-2
13. The CED's enrollment criteria should be revised to secure better candidates.	1	1	0
14. The CED equips its graduates with a life-long ability to develop as experienced teachers after graduation.	0	1	-4
15. The CED provides its students with valuable field training.	0	-2	-3
16. The CED has an outdated curriculum, which is a challenge to graduating distinguished teachers.	-1	-1	4
17. There is a lack of high-quality faculty in the CED which hampers the preparation of teachers.	0	3	3
18. The CED's management impedes the aim of graduating effective teachers.	-3	3	3
19. The CED's students have emotional and psychological needs that are not properly addressed.	1	-1	1
20. The CED's students are prepared with the best practices but face challenges applying these in the government schools.	1	0	-4
21. The CED's students face challenges adopting the college's high standards.	-1	-1	0
***22. The relationship between the CED and the MEHE needs improvement and better collaboration.***	2	4	3
23. There are no problems for students transiting from high school to the CED.	-3	0	-2
24. I think that education is an easy degree at Qatar University, and this hinders teacher education.	-2	1	0
25. The negative view of teaching as a career in Qatar challenges the goal of graduating and recruiting effective teachers.	4	2	-1
***26. As the main national teacher educational provider, the CED is doing excellent work.***	-2	-2	-3
27. The MEHE has an adequate supply of non-Qatari teachers who receive lower salaries than citizens, so there is no interest in preparing Qatari teachers.	-1	-4	1
28. I think the overall culture in Qatar encourages innovation in the teaching profession.	-4	0	-1
***29. I think the Qatari conservative culture drives certain groups to become teachers regardless of their real interest.***	2	2	2

*Italic* statements represent the consensus across the emerging perspectives.
